# Host Plant Availability and Nest-Site Selection of the Social Spider *Stegodyphus dumicola* Pocock, 1898 (Eresidae)

**DOI:** 10.3390/insects13010030

**Published:** 2021-12-27

**Authors:** Clémence Rose, Andreas Schramm, John Irish, Trine Bilde, Tharina L. Bird

**Affiliations:** 1 Department of Biology, Aarhus University, 8000 Aarhus, Denmark; andreas.schramm@bio.au.dk (A.S.); trine.bilde@bio.au.dk (T.B.); tharina@ditsong.org.za (T.L.B.); 2 National Museum of Namibia, Windhoek 1005, Namibia; jirish@biodiversity.org.na; 3 General Entomology Section, Ditsong National Museum of Natural History, Pretoria 0002, South Africa; 4 Department of Zoology and Entomology, University of Pretoria, Private Bag X20, Pretoria 0028, South Africa

**Keywords:** plant-spider interaction, arid environment, microhabitat use, plant structure, survival

## Abstract

**Simple Summary:**

Choosing the right habitat is a critical decision for an animal because it influences its survival and reproduction. Spiders are abundant in all terrestrial habitats including arid habitats. They are often associated with vegetation, which provides structure for building capture webs or activities such as foraging and mating, or which provides shelter and protection. Spiders may select the plant species they live on based on attributes that facilitate these functions. Social spiders live in groups which construct communal silk nests in trees or on shrubs. Little is known about whether and how social spiders choose host plants. In this study, we investigated the use of host plants and the role of host plant features in the social spider *Stegodyphus dumicola* in Namibia. We found that nests were relatively more abundant on specific plant species, on which the spiders also survived better. Spider nests were relatively more abundant on plants higher than 2 m, and on plants with thorns and with a rigid structure. Our findings indicate that social spiders are found more frequently on high and rigid host plants, which provide structure for anchoring their nests and capture webs, and on thorny plants, which may provide protection from browsing animals.

**Abstract:**

An animals’ habitat defines the resources that are available for its use, such as host plants or food sources, and the use of these resources are critical for optimizing fitness. Spiders are abundant in all terrestrial habitats and are often associated with vegetation, which may provide structure for anchoring capture webs, attract insect prey, or provide protective function. Social spiders construct sedentary communal silk nests on host plants, but we know little about whether and how they make nest-site decisions. We examined host plant use in relation to host plant availability in the social spider *Stegodyphus dumicola* Pocock, 1898 (Eresidae) across different arid biomes in Namibia and analysed the role of host plant characteristics (height, spines, scent, sturdiness) on nest occurrence. Host plant communities and densities differed between locations. Spider nests were relatively more abundant on *Acacia* spp., *Boscia foetida*, *Combretum* spp., *Dichrostachys cinerea*, *Parkinsonia africana*, *Tarchonanthus camphoratus*, and *Ziziphus mucronatus*, and nests survived longer on preferred plant genera *Acacia*, *Boscia* and *Combretum*. Spider nests were relatively more abundant on plants higher than 2 m, and on plants with thorns and with a rigid structure. Our results suggest that spiders display differential use of host plant species, and that characteristics such as rigidity and thorns confer benefits such as protection from browsing animals.

## 1. Introduction

The optimal use of resources within an animal’s habitat is critical for maximizing fitness [1,2]. ‘Habitat use’ can be defined as ‘the way an animal uses’ resources of its habitat, for example the use of vegetation or food sources [2]. Patterns of occupancy and resource use are therefore specific to the organism in question [2]. ‘Habitat selection’ refers to the behavioural and decision-making processes involved in making choices of which habitat components to use [2,3]. The selection process results in preferential use of specific habitat components [2,3], for example preference for occupying specific host plant species. Habitat use can affect the fitness of an animal in various ways, for example, through reducing interspecific competition, facilitating maximized feeding activity, and minimizing predation risk [4,5,6,7,8,9,10]. In arid environments, organisms have to deal with extreme ecological conditions such as high temperature fluctuations and low precipitation. These are abiotic factors that may influence daily activity patterns to escape thermal stress and promote physiological responses to avoid dehydration [11,12,13,14,15]. A suitable habitat may also shelter organisms against extreme environmental conditions [16], for example many desert arthropods inhabit burrows [13,17].

Spiders are highly abundant in all terrestrial habitats including arid habitats. Spiders are often associated with vegetation [18], and microhabitat use seems to be primarily determined by differences in vegetation structure. This is manifested in use of different plant structures for activities such as foraging, mating and egg-laying, or functions such as shelter and/or protection for adults and immatures, and nurseries for the offspring [19,20,21,22,23,24]. Individuals may preferentially use specific structures of plants to capture prey (flowers, glandular trichomes) [25], obtain nutrients (nectar, pollen) [26,27,28], obtain protection from predators (thorns) [29] or from desiccation (leaves of bromeliad plants) [20], and to locate mates (flowers) [30]. Spiders may also prefer plant material containing chemical compounds with antimicrobial properties [31], or show preference for specific plants [32,33,34,35], which may result in facultative mutualism with the host plant [36]. For web building spiders, a suitable microhabitat requires substrate to attach the capture web and retreats, offers sufficient prey [4], and provides protection from predators [4]. The sub-social spider *Stegodyphus lineatus* (Latreille, 1817) (Eresidae), for example, tends to reside in tall, spiny or poisonous plants that are rejected by large herbivores and which therefore provide safer nest sites with less disturbances [29]. Protection from web damage may also favour larger webs and thus increased foraging success. Sub-social *Stegodyphus tentoriicola* Purcell, 1904 (Eresidae) spiders that inhabit thorny vegetation are larger and build larger webs than spiders in thornless plants [37]. Plants may increase the foraging success and thus the body size and reproductive success of spiders by attracting prey via flowers, nectar or chemical compounds [38].

Social spiders occur in the tropical environments, and several genera occur in arid environments [39]. Social spiders live in family groups that persist over multiple generations, and individuals cooperate in nest and web maintenance. The spiders build communal silk nests in trees or on shrubs, from which large webs for prey capture extend. The dense silk nest provides protection against biotic and abiotic factors [39]. In contrast to many solitary species that relocate web site in response to biotic or abiotic factors, relocation of nest site in social species is likely to be very costly. Choosing host plants that provide suitable structure for web attachments and protection from web damage is therefore essential. New nests are founded in two ways: (1) by individually dispersing mated females that disperse long distances by ballooning, where they literally take off and fly by aid of a large silk sail that they spin; (2) by individuals that form new nest by colony fission, i.e., individuals walk to disperse a short distance from the natal nest, and initiate a second nest in close proximity to the natal nest [39]. Nest fission is likely to result in nest formation on the same host plant as the natal nest; perhaps this reflects that spiders possess information on the suitability of the host plant given that the nest site has proven successful already, and dispersal over longer distances is costly. Ballooning is likely associated with a high mortality risk, an elevated risk of landing in an unsuitable habitat and of not being able to find and settle on an optimal host plant, making long-distance dispersal highly risky [40,41]. The ability to successfully build a nest on a suitable host plant species is decisive for optimizing fitness, but we know little about whether and how dispersing spiders choose host plants. Host plant traits that influence fitness could be for example flowers that attract insect prey [42], chemical compounds with antimicrobial activity, or features that facilitate web building or provide protection, e.g., thorny plants that protect the nest from destruction by browsing animals [29,43]. Thus, social spiders could develop specific associations with plants that have certain characteristics that benefit them.

A first step to characterize host plant associations with social spiders is to investigate host plant availability and host plant use (occupancy), to test whether there is differential use of host plant species relative to availability. This could be indicative of host plant preference resulting from habitat selection; or, in the absence of any preference, it would indicate random host plant use with differential nest survival among host plants. The social spider, *Stegodyphus dumicola* Pocock, 1898 (Eresidae), is widespread across southern Africa and is found in arid and semi-arid Savanna, grassland and Nama-Karoo habitats [44]. Host plant use in *S. dumicola* in relation to plant species, and whether it is influenced by plant species availability or other characteristics of the host plant remains unknown. We established natural distribution patterns of potential host plants and tested whether plant species predicts spider nest occurrence (i.e., plant use) in different biomes in Namibia. We also analysed relative nest survival in relation to host plant use over a period of two and a half years, to identify adaptive benefits of host plant use. We also asked whether characteristics including plant height, thorns, scent, and sturdiness predict nest occurrence. We sampled data on plant species and characteristics, and spider nest presence at four locations across Namibia. If spiders exhibit differential host plant species use, specific plant species should predict nest occurrence. Alternatively, if plant characteristics (height, thorns, scent, sturdiness) determine host plant use, plants with these characteristics should predict nest occurrence.

## 2. Materials and Methods

### 2.1. Study Species

*Stegodyphus dumicola* is a social spider that lives in family groups in communal nests that they construct on shrubs and trees, or other structures such as human-made fences [39]. Nests consist of one or more brood chambers, and tunnels that link to these brood chambers, and the interior to the outside. One or more three-dimensional capture webs are associated with each nest [39]. Spiders leave the nest only to construct and repair the web at dusk or dawn, or to handle prey caught in the capture web, irrespective of the time of day.

### 2.2. Study Sites and Vegetation Sampling

**Figure 1:**
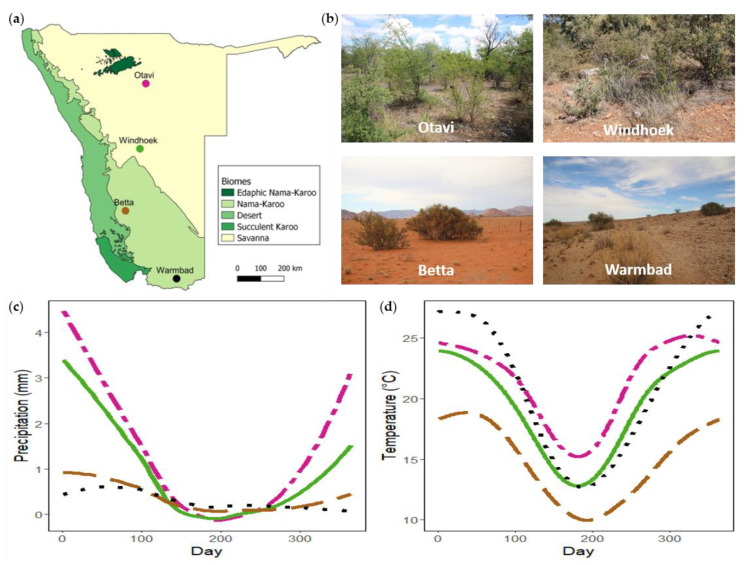
(**a**) Map of Namibia indicating the geographical location of the *Stegodyphus dumicola* Pocock, 1898 study populations with the different biomes (redrawn from [45]). (**b**) Site photos of the four sampling sites. (**c**) Daily average precipitation for each site. (**d**) Daily average temperature for each site.

To determine whether plant species predict nest presence, woody plants were sampled at four populations across Namibia (Figure 1). These populations fall broadly in different biomes (Nama-Karoo and Savanna) (Figure 1a) and are representative of different vegetation types. The Otavi and Windhoek populations fall within the Savanna biome. The Otavi site (−19.47745°, 17.19500°; alt. 1315 m), situated on a farm ~25 km NW of Otavi, represents a broad-leaved Savanna vegetation type (Figure 1b). Common woody species at the site are *Combretum apiculatum, Terminalia prunioides*, *Grewia* spp., *Acacia nebrownii*, and *Dichrostachys cinerea.* The Windhoek site (−22.57012°, 17.21885°; alt. 1923 m), situated in the highlands of Namibia inside a Housing Estate on a semi-nature reserve, represents a fine-leaved Savanna vegetation type (Figure 1b). Woody species common to the site are *Acacia mellifera* and *Tarchonanthus camphoratus.* The Betta and Warmbad populations fall within the Nama-Karoo biome. Vegetation of the Betta site (−25.15323°, 16.24153°; 1211 m) can broadly be described as grassy Nama-Karoo, and of the Warmbad site (−28.44106°, 18.74209°; 987 m) as shrubby Nama-Karoo (Figure 1b). Common woody species at these two sites are *Acacia erioloba, Boscia foetida*, and *Parkinsonia africana*. Both sites are in livestock farming areas, and population sampling were done on the roadside. The populations follow a North-South gradient of annual precipitation and temperature (Figure 1c,d).

Vegetation was sampled using a modified point centred quarter (PCQ) [46] method. At each of the four sites we sampled 10 circular quadrants of 20 m diameter (10 m radius). The centre point of each quadrant was selected based on the current or previous presence of a spider nest—the stem of the plant that contained the nest was taken as the centre point. Within each of the four quarters of each circle, the following parameters were recorded for woody plants: species; abundance of each species; and height of each species according to categories (≤0.5 m, 0.5–2 m, ≥2 m). In each quarter, the number of plants with nests were recorded. Herbaceous plants were not recorded as the nests of social spiders are usually not found on these.

To determine if, and which, chemical or physical cues could influence host plant selection, we made observations on four plant attribute categories and analysed the recorded plant species within each category: (a) Height; (b) Presence/absence of thorns/spines, as these might affect the level of protection against herbivores and predators; (c) Presence/absence of scent, as the latter might influence prey availability or protection against pathogens; and (d) plant structure (rigid or flimsy), as this is likely to determine how sturdy a nest and its capture web are anchored. The presence of aromatic compounds could be in the leaves or in the flowers. Presence of scent or odorous compounds of crushed leaves (*Antiphiona pinnatisecta*, *Croton* spp., *Pechuel–Loeschea leubnitziae*, *Tarchonanthus camphoratus*) [47] and plants presumed to attract large amounts of flying insects during their flowering season based on scent (*Acacia* spp., *Boscia* spp., *Dichrostachys cinerea*, *Grewia* spp., *Olea europaea*, *Searsia* spp.) [47] were classified as “scented plants”. Plants with thorns (*Acacia* spp., *Asparagus* spp., *Sarcocaulon salmoniflorum*, *Ziziphus mucronata*) or with spines (*Carissa bispinosa*, *Combretum imberbe*, *Dichrostachys cinerea*, *Gymnosporia senegalensis*, *Lycium* spp., *Parkinsonia africana*, *Rhigozum trichotomum*, *Terminalia prunioides*, *Ximenia* spp.), were classified as ‘thorns/spines present’ [47]. In the third category, plants with branches that were observed to provide strong resistance when pushed (usually snapping when pushed too far), were classified as ‘rigid’. Those that provided little resistance when pushed down (e.g., *Asparagus* spp., *Euclea* spp.) (Tharina Bird, personal observation) were classified as ‘flimsy’.

### 2.3. Effect of Plant Species and Characteristics on Nest Occurrence

We investigated whether spider nests were distributed randomly on available host plants, or whether there was evidence for preferential use of specific host species or host characteristics on nest occurrence, by analysing the proportion of plant species/plant type (scented; with thorns; structure) with nests present relative to the proportion of plants/plant types available in the habitat. All analyses were performed in R software, version 4.0.5 [48]. We ran a chi-square test with Yates correction to test the null hypothesis that nest presence is independent of plant species and characteristics (height, scent, with thorns, structure). For plant species, we ran the test for each population because populations differed in plant abundance and diversity (Table S1). For plant characteristics, we ran the test on the overall dataset.

### 2.4. Survival Analysis

To determine whether nest survival depends on plant species, we used an unpublished data set originating from a study that investigated the *S. dumicola* microbiome [49]. Data on nest survival and host plant species were collected in Namibia every three months over a two-and a half years period. Spiders from the same nests were sampled for microbiome analysis at each time point, which implies that each nest was revisited every 3 months, providing information on nest longevity. For each nest, the host plant species (tree or shrub that held the nest) and the presence of live spiders as the criteria for whether a nest was ‘dead’ or ‘alive’ was recorded, allowing us to assess nest longevity depending on host plant species. A nest that has ‘died’ quickly deteriorates, capture webs are destroyed, and no live spiders are present. Note that spider nests included in this analysis were already established at the time they were included in the monitoring scheme. We therefore do not know the absolute survival time of each nest. Our analyses therefore include relative survival time in days from the starting point of the observations and until the nest died.

We ran a Kaplan-Meier analysis on a data set of 38 nests. We pooled the plants to genus level to ensure a sufficiently high sample size per genus: *Acacia* spp. (*n* = 18), *Boscia* spp. (*n* = 8), *Combretum* spp. (*n* = 6), *Dichrostachys cinerea* (*n* = 6). We ran log rank tests to compare nest survival times between genera followed by pairwise comparisons with Bonferroni correction when differences were significant. Nest survival was also estimated for each population (Otavi *n* = 14, Windhoek *n* = 9, Betta *n* = 8, Warmbad *n* = 7). The analysis was run in R using the package “survival” v.3.2-10 [50] and plotted using “survminer” v0.4.9 [51]. Nests still alive at the end of the study were right censored.

## 3. Results

### 3.1. Plant Species, Height and Characteristics Influence Nest Presence

**Figure 2:**
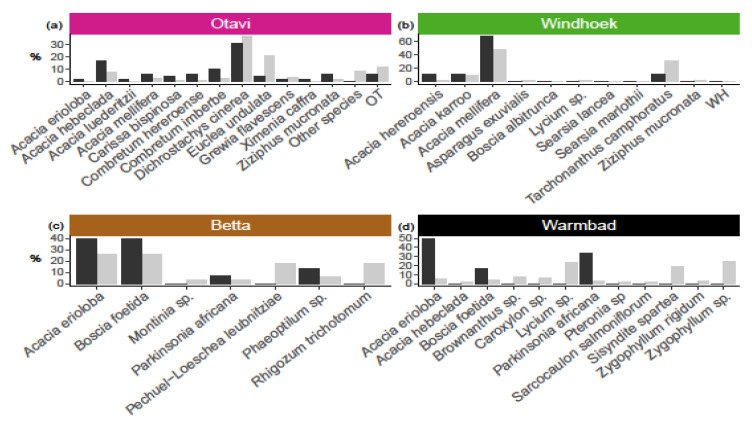
Plant species influence on presence of social spider *Stegodyphus dumicola* Pocock, 1898 nest(s), as indicated by abundance of plant species (grey bars; given as percentage of all plants species in the population), versus abundance, for each plant species, of plants with nest (black bars; given as percentage of all plants with nest), in four populations (**a**) Otavi, (**b**) Windhoek, (**c**) Betta, (**d**) Warmbad. Data show that nest presence was affected by plant species in Otavi (χ^2^ = 106.84, df = 39, *p* < 0.001), Betta (χ^2^ = 16.625, df = 6, *p* < 0.01) and Warmbad (χ^2^ = 54.99, df = 11, *p* < 0.001), but with no effect detected in Windhoek (χ^2^ = 4.68, df = 11, *p* = 0.95). For Otavi, only plant species with nests are shown, (apart from the category OT=other species); see Table S1 for the complete list of plants and sample sizes. OT and WH are unknown plant species from Otavi and Windhoek respectively.

At Otavi, we found relatively more nests on *Acacia hebeclada*, *A. mellifera*, *Combretum hereroense*, *C. imberbe*, *Dichrostachys cinerea* and *Ziziphus mucronata* (Figure 2b), and nest presence was affected by plant species (χ^2^ = 106.84, df = 39, *p* < 0.001). At Betta and Warmbad, most of the nests were found on *Acacia erioloba*, *Boscia foetida* and *Parkinsonia africana* (Figure 2c,d), and again we detected a non-random pattern of nest occurrence (Betta: χ^2^ = 16.625, df = 6, *p* < 0.01, and Warmbad: χ^2^ = 54.99, df = 11, *p* < 0.001). At Windhoek, we found nests only on *Acacia hereroensis*, *A. karroo*, *A. mellifera* and *Tarchonanthus camphoratus*, which are the main plants that are available (Figure 2b). The nest occurrence pattern in Windhoek therefore reflects the plant species distribution (χ^2^ = 4.68, df = 11, *p* = 0.95).

**Figure 3:**
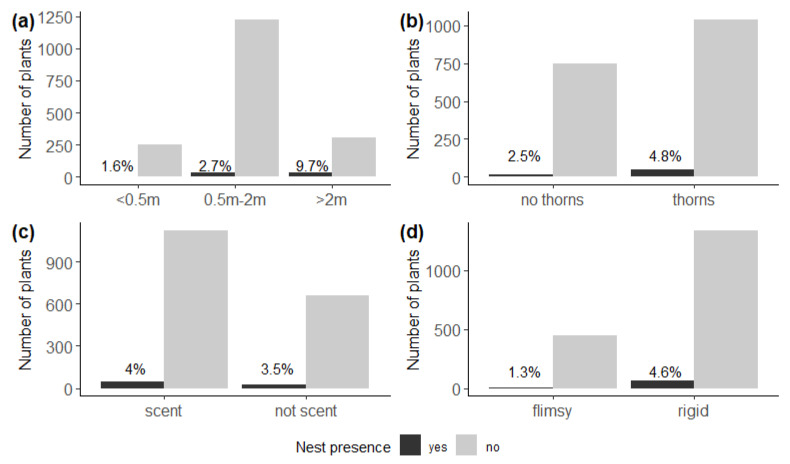
Number of potential host plants with (black) and without (grey) nests of *Stegodyphus dumicola* Pocock, 1898, according to (**a**) plant height, indicating more nests on plants higher than 2 m (χ^2^ = 39.6, df = 2, *p* < 0.001); (**b**) spinosity, indicating more nests on host plants with thorns (χ^2^ = 5.72, df = 1, *p* = 0.02); (**c**) scent, indicating no effect of odour or scent on nest presence (χ^2^ = 0.18, df = 1, *p* = 0.67); and (**d**) structure, indicating more nests on plants with a woody rather than flimsy structure (χ^2^ = 9.23, df = 1, *p* < 0.01). Percentages indicate the proportion of host plants with a nest.

We found relatively more nests on plants higher than 2 m (χ^2^ = 39.6, df = 2, *p* < 0.001) (Figure 3b), and on host plants with thorns (χ^2^ = 5.72, df = 1, *p* = 0.02) (Figure 3b), and also on plants with a woody rather than flimsy structure (χ^2^ = 9.23, df = 1, *p* < 0.01) (Figure 3d). We detected no effect of scent on nest presence (χ^2^ = 0.18, df = 1, *p* = 0.67) (Figure 3c).

### 3.2. Nest Survival Rate Differs between Nesting Plant Genera and Populations

**Figure 4:**
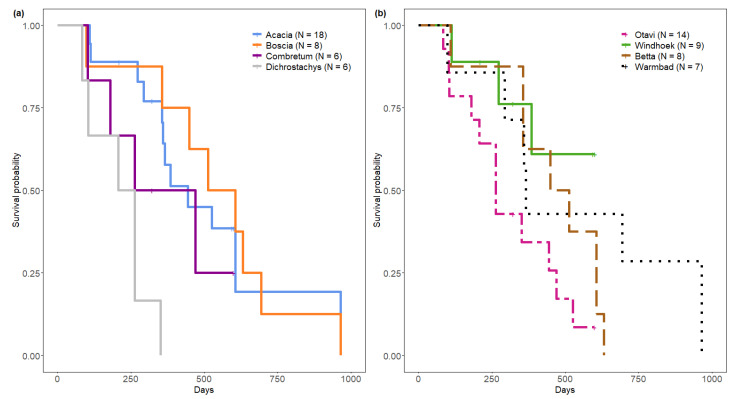
Kaplan Meier survival analysis of *Stegodyphus dumicola* Pocock, 1898 nests, t0 = sampling start. (**a**) Nest survival per nesting plant genus. (**b**) Nest survival per population.

Nests differed in their survival rate according to plant genus (log rank test, χ^2^ = 16.8, df = 3, *p* < 0.001) (Figure 4b). Median survival time from sampling start was 234, 366, 443 and 559 days for nests on *Dichrostachys cinerea*, *Combretum* spp., *Acacia* spp. and *Boscia* spp. respectively. Nest survival rate was significantly lower on *Dichrostachys cinerea* compared to nests on *Acacia* spp. (*p* < 0.001) and on *Boscia* spp. (*p* < 0.01), but not compared to nests on *Combretum* spp. (*p* = 0.31). We detected no differences in nest survival rate on *Boscia* spp., *Acacia* spp. and *Combretum* spp.

Populations differed in nest survival (log rank test, χ^2^ = 8.2, df = 3, *p* = 0.04). Median survival time from sampling start was 263 days for Otavi, 480 days for Betta and 365 days in Warmbad, with the overall longest survival in Warmbad (Figure 4b).

Windhoek’s median survival time could not be estimated for the full study period as this population was observed for a shorter time period, which was too short to detect the 50% survival threshold (Figure 4b).

## 4. Discussion

In this study, we investigated host plant use in the social spider *S. dumicola*, based on plant species composition and availability, together with different characteristics of host plants. We found some evidence that plant species predicted spider nest occurrence, and that host plant use differed among populations living under different ecological conditions in different biomes. The most frequently used host plants were *Acacia* spp., *Boscia foetida*, *Combretum* spp., *Dichrostachys cinerea*, *Parkinsonia africana*, *Tarchonanthus camphoratus*, and *Ziziphus mucronata*. These host plants contained relatively more spider nests than predicted by their occurrence alone. This pattern would be consistent with the exhibition of preference for certain host plant species. Alternatively, the result could reflect a random distribution of nests combined with differential survival on different host plants. However, if there is differential nest survival among host plants, nest-site selection would be adaptive, and we would expect *S. dumicola* to develop the ability to actively choose a superior host plant.

Spiders may use structural features of the habitat as cues for settling in a site [4], and vegetation structure may influence microhabitat use [19]. Our data suggests that plant characteristics such as height, presence of thorns, and sturdiness influence nest occurrence. We found that spider nests occurred more frequently on species higher than 2 m, and on thorny plants. This is consistent with the hypothesis that the presence of thorns or spines may increase nest survival through protection against bird predation or destruction from browsing animals, or alternatively increase prey capture through improved web-attachment possibilities [37]. These benefits could potentially be magnified in larger and taller host trees, for example through enlarged three-dimensional structure and rigidity, or relatively higher production of flowers. Indeed, we found that sturdiness (rigid compared with flimsy) predicted the occurrence of spider nests, as would be expected if woody plants provide better protection or structural advantages. It is also possible, that females are simply more likely to land in larger trees due to their larger coverage.

Higher and more sturdy plants are typically also older plants, which have been under selection to survive in arid environments. For example, older and larger *Artemisia ordosica* plants are able to withstand severe drought stress (70% rainfall reduction) better than smaller and younger *A. ordosica* [52]. Larger and more sturdy plants are therefore less likely to perish under drought, which would simultaneously cause extinction of any spider nests located on them. Alternatively, the death of the host plant may force spiders to attempt to relocate, which is associated with high risk of mortality [53]. Collectively, our findings raise the question of whether the observed patterns of host plant use represent active choices, or simply differential survival of different host plants. While the latter is expected to exert selection on spiders to develop active host plant choices, it remains challenging to identify causal relationships in observational studies. We performed a survival analysis, which provides some insights into the fitness consequences of host plant use in *S. dumicola*. Although this analysis was based on genera and not species, due to the low representation of nests across species, we found improved nest survival on *Boscia* spp., *Combretum* spp. and *Acacia* spp. respectively, which are the genera that also contain the preferred host species. Note that nests were observed for a period of two and a half years, and their full lifespan was not known, therefore this result is tentative.

It has been suggested that vegetation structure and dispersal mode influence spatial distribution patterns of *S. dumicola* nests [54]. The presence of multiple nests on the same host plant typically results from fission of the natal nest [39], generating an association between presence of the natal nest and establishment of a new nest, which may reflect the previous success of the natal nest and provide information on the suitability of the host plant. We found more than one nest in 21 out of 71 trees with nests, of these 9 were on *Acacia* spp., 4 on *Boscia foetida*, 3 on *Parkinsonia africana*, and 2 each on *Combretum imberbe* and *Ziziphus mucronata.* These are also the host plants with highest occurrence of nests overall, and nests on *Boscia, Acacia*, and *Combretum* had the highest survival. If ballooning spiders exhibit any form of host plant choice, the presence or absence of an existing nest could further influence this choice. Given that nests depend on the arrival of insect prey into their capture webs, and that resource competition for prey increases with nest size [55], it seems reasonable to suggest that existing spider nests should deter dispersers from establishing a new nest in its immediate vicinity to avoid competition for prey. Indeed, natal nests are often found to go extinct while the new nest established by fission on the same host plant is active, perhaps due to competition for prey (T.L.B. and T.B., personal information).

The availability and density of suitable host plants will influence and potentially limit the opportunities for a ballooning female to exert host plant choice. Our study revealed differences in plant species composition among the collection sites, consistent with their location in different biomes. For example, there were fewer host plants available in Betta and Warmbad compared with Otavi and Windhoek. This suggests that *S. dumicola* may have to employ opportunistic strategies in host plant use. Nevertheless, our results show non-random use of host plant species, but also that host plant use varies between sites, which in turn vary in host plant availability. This suggests a degree of generalism and resilience that might be adaptive by enabling populations to occupy different arid habitats, which vary in environmental and climatic conditions.

We also investigated whether scented plants are more likely to host spider nests, based on the assumption that certain chemical compounds produced by the host plant might provide direct or indirect benefits to the spiders, e.g., by attracting prey, or through the production of antimicrobial compounds [31]. For example, the jumping spider *Lyssomanes viridis* (Walckenaer, 1837) exhibits a chemically mediated preference for the plant *Liquidambar styraciflua* [31], which contains volatile broad-spectrum antimicrobial compounds [56]. The spider experiences higher hatching success on this plant than on other sympatric species or substrates [31]. We did not detect plant use based on scented traits; however, it is important to note here that our categorisation of scented plants is very broad and does not permit resolution of more specific compounds such as phenolics, terpenes, or flavanoids, many of which are plant species-specific [57]. Nevertheless, there are some promising ideas for how and why specific host plant chemical compounds might play a role in plant-spider interactions. The *S. dumicola* nest is made predominantly of silk, into which plant material and sometimes exoskeletons of prey is incorporated, and the nest hosts a unique microbiome [58]. A recent study showed that *S. dumicola* nests (the actual silk retreat) contain volatile organic compounds with antimicrobial properties, which may protect the spiders against pathogens; many of these volatiles likely originated from the host plants [59]. The nest microbiome and associated volatile compounds are influenced by the local environment, notably by the plants on which the spiders build their nests [59]. In our study, we found relatively more nests on *Acacia mellifera* and *Combretum imberbe*, which are both known for producing antimicrobial compounds [60,61,62,63]. Alternatively, the host plant could facilitate beneficial conditions for microorganisms that produce antimicrobial compounds or other metabolites beneficial to the spiders [58,59,64]. This poses the interesting question of whether spiders can acquire particular beneficial microbial nest symbionts by choice of host plant, or by integrating specific plant material into the nest.

The survival analysis recovered variation in nest survival among locations. Between populations, median nest longevity ranged between 263–365 days. The longest nest survival that we recorded was ~950 days (or just over 2.5 years). This is in accordance with high nest turnover rates in *S. dumicola* [65] with less than 10% of first generation nests surviving a single generation [53]. Longevity of the social congener *S. mimosarum* was on average 16 months, with relatively few nests surviving more than 2-3 annual generations [66]. Interestingly, the lowest survival was found in Otavi, which is the most productive habitat with higher plant abundance and species richness. The Otavi location also contained relatively more spider nests, consistent with macro-ecological analysis showing that habitat productivity positively influences social spider distribution [67,68]. The lower nest survival rate in Otavi is therefore counter intuitive. One possible explanation for this is that the prevalence of pathogenic fungi is higher in the more humid Savanna habitat of Otavi [11,49], accelerating nest mortality. It was previously proposed that fungal infections are more severe under higher humidities [65], and indeed fungi are more prevalent in Otavi [58]. These findings suggests that variation in host plant availability, host plant quality and climatic conditions on the one hand, and pathogenic microorganisms on the other hand, likely generate opposing selection pressures on the survival of the social spider *S. dumicola* in different geographical locations. We propose that these complex and dynamic interactions shape the wide distribution of *S. dumicola* across arid environments.

## 5. Conclusions

The social spider *S. dumicola* exhibits differences in host plant use between sites, which differ in host plant availability and density. The most frequently used host plants were *Acacia* spp., *Boscia foetida*, *Combretum* spp., *Dichrostachys cinerea*, *Parkinsonia africana*, *Tarchonanthus camphoratus*, and *Ziziphus mucronata*. We found improved nest survival on *Boscia* spp., *Combretum* spp. and *Acacia* spp. respectively, suggesting that differential use of host plants might be associated with fitness benefits to the spiders. Experimental studies are required to assess whether the spiders exhibit active choice of host plant for their nest site. Nests were relatively more abundant on plants higher than 2 m, and on plants with thorns and with a rigid structure, indicating that spiders may differentially use plants that provide benefits such as protection against web destruction from browsing animals.

## Data Availability

The data presented in this study are available in Supplementary Material.

## Supplementary Materials

The following are available online at https://www.mdpi.com/article/10.3390/insects13010030/s1, Table S1. Proportion of potential host plant species (Plant) and *Stegodyphus dumicola* Pocock, 1898 nests (Nest) for each plant species, per study site. Empty cells indicate that the species was not found in the study sites. Total sample size (Sample size line) is given both for the number of plants (Plants (N=)) and the number of plants with a nest (Nests (N=)) for each study site. OT = unknown host plant species in Otavi, WH = unknown host plant species in Windhoek.
